# The role of nutrition in promoting growth in pre-term infants with bronchopulmonary dysplasia: a prospective non-randomised interventional cohort study

**DOI:** 10.1186/1471-2431-14-235

**Published:** 2014-09-22

**Authors:** Maria Lorella Giannì, Paola Roggero, Maria Rosa Colnaghi, Pasqua Piemontese, Orsola Amato, Anna Orsi, Laura Morlacchi, Fabio Mosca

**Affiliations:** NICU, Department of Clinical Sciences and Community Health, Fondazione IRCCS Cà Granda Ospedale Maggiore Policlinico, Università degli Studi di Milano, Via Commenda 12, 20122 Milan, Italy

**Keywords:** Pre-term infants, Bronchopulmonary dysplasia, Growth, Nutrition

## Abstract

**Background:**

Pre-term infants who develop bronchopulmonary dysplasia (BPD) are at risk of postnatal growth failure. It has been reported that energy expenditure is higher in infants with BPD than in those without BPD. The aim of the study was to evaluate whether increasing the enteral energy intake of pre-term infants with BPD can improve post-natal growth.

**Methods:**

This prospective, non-randomised interventional cohort study was designed to assess growth in 57 preterm infants with BPD (gestational age <32 weeks, birth weight <1500 g, and persistent oxygen dependency for up to 28 days of life) fed individually tailored fortified breast milk and/or preterm formula, and a historical control group of 73 pre-term infants with BPD fed breast milk fortified in accordance with the instructions of the manufacturer and/or pre-term formula. Between-group differences in the continuous variables were analysed using Student’s *t* test or the Mann-Whitney test; the discrete variables were compared using the chi-squared test. Linear regression analysis was used to investigate the independent contribution of enteral energy intake to weight gain velocity.

**Results:**

The duration of parenteral nutrition was similar in the historical and intervention groups (43.7 ± 30.9 *vs* 39.6 ± 17.4 days). After the withdrawal of parenteral nutrition, enteral energy intake was higher in the infants in the intervention group with mild or moderate BPD (131 ± 6.3 *vs* 111 ± 4.6 kcal/kg/day; p < 0.0001) and in those with severe BPD (126 ± 5.3 *vs* 105 ± 5.1 kcal/kg/day; p < 0.0001), whereas enteral protein intake was similar (3.2 ± 0.27 *vs* 3.1 ± 0.23 g/kg/day).

Weight gain velocity was greater in the infants in the intervention group with mild or moderate BPD (14.7 ± 1.38 vs 11.5 ± 2 g/kg/day, p < 0.0001) and in those with severe BPD (11.9 ± 2.9 vs 8.9 ± 2.3 g/kg/day; p < 0.007). The percentage of infants with post-natal growth retardation at 36 weeks of gestational age was higher in the historical group (75.3 vs 47.4; p = 0.02).

**Conclusions:**

On the basis of the above findings, it seems that improved nutritional management promotes post-natal ponderal growth in pre-term infants with BPD.

**Electronic supplementary material:**

The online version of this article (doi:10.1186/1471-2431-14-235) contains supplementary material, which is available to authorized users.

## Background

Bronchopulmonary dysplasia (BPD) is one of the most frequent morbidities affecting extremely pre-term infants. Despite advances in medical and respiratory care, the incidence of BPD has not decreased but remains about 42% in infants born at a gestational age of 22-28 weeks [1].

Adequate nutrition plays a major role in modulating lung development and maturation [2], and it has been demonstrated that under-nutrition exacerbates the alveolar damage caused by hyperoxia in experimental models and contributes to the development of emphysema in humans [3, 4].

It is difficult to ensure adequate nutritional support in pre-term infants with BPD because of their increased respiratory needs and the occurrence of chronic lung injury [5]. Furthermore, a worsening in their respiratory status frequently interrupts progress to full enteral nutrition because of more frequent feeding intolerance or clinicians’ concerns about aggressively increasing enteral feeds. Consequently, the pre-term infants who develop BPD are at high risk of post-natal growth failure [6].

Wemhoner et al. [7] have recently reported that a critical amount of enteral supply is essential for promoting lung development and preventing BPD. They found that pre-term infants who were subsequently diagnosed as having BPD received less enteral protein and energy during the first 14 days of life than infants who did not develop BPD. It has also been reported that energy expenditure is higher in infants with BPD than in those without [8, 9], and so the former may require a higher energy intake in order to achieve sustained growth.

Although there are very few studies that have investigated protein requirements in infants with BPD [5], it can be assumed that their protein needs are similar to those of infants without BPD. However, Huysman et al. [10] found a lack of fat-free mass six weeks post-term in pre-term infants with BPD, thus suggesting that their protein intake may be inadequate. It is widely acknowledged that it is important to give infants with BDP calcium, phosphorus and vitamin D supplements in order to prevent the occurrence of rickets and promote fat-free mass deposition [5, 11]. Supplementation with other nutrients such as vitamin A, vitamin E and inositol has also been investigated, but there there is no clear evidence supporting their routine use in the nutritional management of infants with BPD [5, 11].

In order to test the hypothesis that preterm infants developing BPD would show greater weight gain velocity if their enteral energy intake was higher than that of a historical group of pre-term infants with BPD, the primary aim of this study was to evaluate whether increasing the enteral energy intake of pre-term infants with BPD modulates post-natal growth. The secondary aim was to investigate the independent contribution of enteral energy intake to weight gain velocity in preterm infants with BPD receiving enteral nutrition.

## Methods

This prospective, non randomised interventional cohort study was approved by the institutional Ethics Committee of the Fondazione Istituto di Ricovero e Cura a Carattere Scientifico Cà Granda Ospedale Maggiore Policlinico, Milan (Italy), and informed written consent was obtained from the parents of the participants. The study are reported following the STROBE guidelines for cohort studies (for more details see Additional file 1).

### Subjects

The intervention group consisted of infants admitted to our Institution between January and December 2013, who were compared with a historical group of infants admitted between January 2011 and December 2012. The inclusion criteria were a birth weight of <1500 g, a gestational age of <32 weeks, and persistent oxygen dependency for up to 28 days of life; the exclusion criteria were death during hospitalisation, early transferred to other units, and the presence of congenital anomalies.

The recorded neonatal data were gestational age at birth; birth weight; body length and head circumference; gender; singleton pregnancy; Apgar scores at 1′ and 5′; the need of tube feeding at discharge. The parameters recorded at 36 weeks of gestational age were the severity of bronchopulmonary dysplasia; the presence of post-natal growth retardation; body weight and length, and head circumference. The medications recorded included pre- and post-natal steroids, artificial surfactants and diuretics. A record was also made of the occurrence of patent ductus arteriosus, surgical necrotising enterocolitis, intraventricular hemorrhage, retinopathy of prematurity and sepsis, defined as the presence of a positive blood culture. Respiratory support was recorded as the number of days of assisted ventilation and the number of days of supplemental oxygen therapy at an FiO2 value >0.21.

Gestational age was based on the last menstrual period and first-trimester ultrasonogram. The infants with a birth weight in the <10th or ≥10th percentile for gestational age on the basis of Fenton’s growth chart [12], were respectively classified as having a weight that was small for gestational age (SGA) or appropriate for gestational age (AGA). BDP was defined as mild, moderate or severe on the basis of the classification of Jobe and Bancalari [13]. Post-natal growth retardation was defined as a weight at 36 weeks of gestational age that was in the <10th percentile for gestational age on the basis of Fenton’s growth chart [12].

### Nutritional regimens

The parenteral solutions were prepared by the hospital pharmacy in accordance with medical prescriptions. They provided a minimum of 57 kcal/kg/day with 2.5 g/kg of proteins on the first day of life, and up to 90–100 kcal/kg/day and 4 g/kg/day of proteins within the first week. Enteral feeding was started within 24 hours of post-natal life using breast milk or pre-term formula in the absence of breast milk (energy 83 kcal/100 mL; carbohydrates 8.4 g/100 mL; proteins 2.9 g/100 mL; fat 4.1 g/100 mL). Weaning from parenteral nutrition was scheduled in order to obtain a weight gain velocity of ≥15 g/kg/day, with the parenteral provision of macronutrients (especially proteins) being gradually reduced and the intake of enteral macronutrients increased on the basis of the infant’s weight gain velocity [14].

The nutritional intervention focused on enteral nutrition. When tolerated, an enteral intake of >100 mL/kg of individually tailored fortified breast milk and/or pre-term formula (in the case of no or insufficient breast milk) was given with the addition of Duocal (Nutricia, Germany) and MCT oil (Nestlé, Switzerland) in order to reach a mean energy intake of between 120 and 150 kcal/kg/day. Carbohydrates and fat respectively provided 50% and 35% of the total energy intake. In addition, FM 85 (Nestlé) and Protifar (Nutricia) were used to ensure a mean protein intake of ≥3.5 g/kg/day.

In the historical control group, breast milk was fortified as indicated by the manufacturer (FM 85: 5 g/100 mL of breast milk) when the infants tolerated an enteral intake of >100 ml/kg/day. In the absence of breast milk or when breast milk was insufficient, the infants were fed a pre-term formula.

The infants in both groups received similar supplementations of calcium (140 mg/kg/day), phosphorus (90 mg/kg/day) and vitamin D (800 IU/day).

### Nutritional and growth data

Energy and protein enteral intakes were calculated daily from the patients’ computerised medical charts.

### Growth measurements

Growth was assessed by two medical investigators. Daily body weight and weekly body length and head circumference were measured using standard procedures [15]. Body mass was measured to the nearest 0.1 g using a precise electronic scale and body length to the nearest 1 mm using a Harpenden neonatometer (Holtain Ltd., UK), and head circumference to the nearest 1 mm using a non-stretch measuring tape. Weight gain velocity was assessed using the formula: [1000xln (Wn/W1)]/(Dn-D1), in which W = weight in grams; D = day; 1 = beginning of the time interval n = the end of the time interval [16].

### Statistical analysis

The date are expressed as mean values and standard deviations or the number and percentage of observations. Between-group differences in growth parameters, and energy and protein intakes were analysed using Student’s *t* test or the Mann-Whitney test; the chi-squared test was used to compare the discrete variables.

In order to investigate the independent contribution of enteral energy intake to weight gain velocity while controlling for birth weight, gestational age, being a twin, gender, the severity of BPD, the occurrence of co-morbidities (patent ductus arteriosus, surgical necrotising enterocolitis, intraventricular hemorrhage, retinopathy of prematurity, and sepsis), diuretic therapy and enteral protein intake, linear regression analysis was used to analyse the pooled data of the infants in each group.

Statistical significance was set at a p value of 0.05 level. All of the statistical analyses were made using SPSS software, version 12 (SPSS Inc., Chicago, IL).

## Results

The study involved a total of 130 infants, whose basic characteristics are shown in Table 1. There was no statistically significant between-group difference in the subjects’ basic characteristics, the occurrence of comorbidities or the medications received during hospitalisation (except for diuretics, which were administered to a higher percentage of infants in the intervention group). Similar percentages of infants in the intervention and control group were fed exclusively fortified breast milk (14% vs 17%), exclusively pre-term formula (28% vs 26%), or both (57% vs 56).

**Table 1:** **Descriptive data**

	Intervention group (n = 57)	Historical group (n = 73)
Birth weight, g	852 ± 253	854 ± 240
Gestational age, weeks	26 ± 2.2	26 ± 2.2
Males, n (%)	30 (52.6)	40 (54.8)
SGA, n (%)	15 (26.3)	19 (26.0)
Twins, n (%)	19 (33.3)	30 (41.1)
Apgar 1′	5.1 (1.8)	5.0 (1.9)
Apgar 5′	7.8 (0.9)	7.8 (1.05)
Infants receiving artificial surfactant, n (%)	50 (87.7)	63 (86.3)
Infants treated with prenatal steroids, n (%)	45 (78.9)	52 (71.2)
Infants treated with postnatal steroids, n (%)	19 (33)	14 (19.2)
Infants receiving diuretic therapy, n (%)	47 (82.5)*	33 (45.2)
Days of assisted ventilation	41.26 (51.13)	36.5(37.4)
Days of supplemental oxygen therapy	25.8 (26.1)	29.1 (30.7)
Mild and moderate BPD, n (%)	36 (63.2)	48 (65.7)
Severe BPD, n (%)	21 (36.8)	25 (34.2)
Patent ductus arteriosus, n (%)	36 (63.2)	43 (58.9)
Necrotizing enterocolitis, n (%)	2 (3.5)	3 (4.1)
Intraventricular hemorrhage, n (%)	10 (17.5)	19 (26.0)
Retinopathy of prematurity, n (%)	21 (36.8)	21 (28.8)
Sepsis, n (%)	7 (12.3)	15 (20.5)
Infants with tube feeding at discharge, n (%)	7 (9.6)	3 (5.3)

*p = <0.0001.

Mean values ± standard deviation, or absolute numbers (%).

*SGA* small for gestational age; *BPD* bronchopulmonary dysplasia.

Table 2 shows the anthropometric parameters at 36 weeks of gestational age. Weight was significantly higher in the intervention group, and the percentage of infants with post-natal growth retardation was significantly higher in the historical control group.

**Table 2:** **Anthropometric parameters at 36 weeks of gestational age**

	Intervention group (n = 57)	Historical group (n = 73)
Weight, g	1957 ± 402*	1841 ± 317
Length, cm	41.1 ± 3.7	40.6 ± 3.2
Head circumference, cm	29.6 ± 1.7	29.4 ± 1.9
Postnatal growth retardation, n (%)	27 (47.4)**	55 (75.3)

*p = 0.04; **p = 0.02.

Mean values ± standard deviation, or absolute numbers (%).

The mean duration of parenteral nutrition was similar in the historical and intervention groups (43.7 ± 30.9 vs 39.6 ± 17.4 days; p = 0.8). After the withdrawal of parenteral nutrition, mean enteral energy intake was significantly higher in the intervention group, whereas there was no between-group difference in enteral protein intake. Weight gain velocity during enteral nutrition was significantly higher in the intervention group (Table 3).

**Table 3:** **Enteral nutritional data**

	Intervention group (n = 57)	Historical group (n = 73)
Volume intake, mL/kg/day	142.5 ± 8.7	140.9 ± 8.5
Protein intake, g/kg/day	3.2 ± 0.27	3.1 ± 0.23
***Mild and moderate BPD***		
Energy intake, kcal/kg/day	131 ± 6.3**	111 ± 4.6
Weight gain velocity, g/kg/d	14.7 ± 1.38**	11.5 ± 2
**Severe BPD**		
Energy intake, kcal/kg/day	126 ± 5.3**	105 ± 5.1
Weight gain velocity, g/kg/d	11.9 ± 2.9*	8.9 ± 2.3

*p = 0.007; **p < 0.0001.

Mean values ± standard deviation.

*BPD* bronchopulmonary dysplasia.

Regression analysis (R2 = 0.538, p < 0.0001) showed that enteral energy intake was positively associated with weight gain velocity during enteral nutrition (unstandardised B coefficient 0.127), and inversely with the severity of BDP (unstandardised B coefficient -2.92) and the occurrence of necrotising enterocolitis (unstandardised B coefficient -3.27).

## Discussion

The findings of this study indicate that increasing the enteral energy intake of pre-term infants with mild and moderate BPD together with the provision of a protein intake of 3 g/kg/day leads to a mean weight gain velocity of 14.7 g/kg/day, that approximates the fetal growth rate recommended by the American Academy of Pediatrics [17]. In addition, the percentage of infants discharged with post-natal retardation at 36 weeks of gestational age was significantly lower in the intervention group. On the other hand, the infants with severe BPD did not achieve the same weight gain velocity during enteral nutrition, probably because of their extremely high respiratory needs [11]. Linear regression analysis showed that enteral energy intake, the severity of BPD and the occurrence of necrotising enterocolitis were the main determinants of weight gain velocity during enteral nutrition. There was no significant between-group difference in the percentage of infants receiving post-natal steroids or in the number of days of assisted ventilation or supplemental oxygen therapy. It can be speculated that the significantly higher percentage of infants receiving diuretic therapy in the intervention group was incidental as the severity of BDP was similar in the two groups.

The greater weight gain velocity in the infants fed an increased enteral energy intake may be partially explained by the fact that infants with BPD require more energy to support the increased work load involved in breathing and maintaining metabolic rate [5, 9]. We increased energy intake as it has been found that infants with BPD need more calories than age-matched healthy controls [11]. However, it must be noted that, although mean actual energy intake ranged from 126 to 131 kcal/kg/day, BPD-related feeding difficulties prevented higher energy intakes that may have promoted growth in the most severely affected infants. As suggested by others [11], in order to meet further caloric demands, we used fat rather than carbohydrates because this allows energy supplementation using small volumes and produces low amounts of carbon dioxide. Furthermore, fat was increased rather than protein intake in order to avoid protein oxidation and promote tissue accretion [18].

Although there are very few published data, it is assumed that the proteins needs of infants with or without BPD are similar [5]. However it has been suggested that infants with BDP might have a greater need for proteins because their relative lack of fat-free mass probably reflects inadequate protein intake [10]. We could not reach our targeted protein intake because of the occurrence of episodes of feeding intolerance, and so actual protein intake was only 3.2 g/kg/day, which is insufficient to meet the protein requirements of especially the most severely affected infants.

It is widely acknowledged that calcium, phosphorus and vitamin D supplementation is extremely important in order to promote bone mineralisation in pre-term infants and prevent the occurrence of osteopenia of prematurity [5, 11], and the infants in both groups received similar supplementations of these nutrients. We decided not to give the infants in the intervention group vitamin A or vitamin E supplements, or supplements of other micronutrients such as inositol and selenium because of the lack of any clear evidence that their routine use is beneficial in infants with BDP [5, 11].

Our results are consistent with previously published data [19, 20] indicating that improved nutritional strategies can positively affect the growth of pre-term infants with BPD, who frequently suffer from post-natal growth retardation [6, 21]. Madden et al. [19] found a significant increase in mean weight z-scores and a decrease in the proportion of infants with a mean weight z-score of <2 SD at 20 months of corrected age in a cohort of extremely low gestational age infants with BPD born in 2000-2003 in comparison with a cohort of extremely low gestational age infants with BPD born in 1996-1999. The authors speculated that the improvement in the growth outcomes may have been due to changes in the infants’ nutritional management, such as the use of more aggressive parenteral nutrition and more caloric post-discharge formulae. Theile et al. [20] retrospectively studied 88 extremely low birth weight infants with BPD, and found a reduction in post-natal growth retardation at the time of hospital discharge (as assessed on the basis of weight and head circumference) in infants fed caloric-dense milk. However, as we found that the improved weight gain in the infants belonging to the intervention group was not associated with an improvement in length gain, there may be some concern about the quality of their growth because, although accompanied by the provision of a sufficient protein intake, the increased caloric intake may have caused relatively greater fat deposition [22]. On the other hand, as it has been reported that infants with BDP show a lack of both fat-free mass and fat mass deposition [10, 23], it can also be speculated that the high energy intake may have allowed at least a partial recovery of fat mass and that the recovery of fat-free mass may occur later. Furthermore, the increased caloric intake may have been used to meet the high metabolic requirements of infants affected by BDP, thus allowing the use of proteins for lean tissue accretion and preventing protein oxidation.

The promotion of adequate ponderal growth is of major importance as it has been associated with a favourable neurodevelopment outcome throughout childhood [24, 25]. It is also agreed that BPD has an unfavourable effect on the cognitive outcome of children born very pre-term that seems to persist into school age even in the absence of severe brain lesions [26].

This clinically interesting study has three limitations. First of all, as it was a prospective non- randomised interventional cohort study, it may have been affected by a higher number of known and unknown confounders than a randomised, controlled trial; secondly, the number of enrolled infants was relatively small; and thirdly, the infants’ body composition was not assessed.

## Conclusions

The findings of this study suggest using nutritional supplements to increase enteral energy intake in association with an adequate protein intake promotes post-natal ponderal growth in pre-term infants affected by BPD. However, further studies are required in order to investigate the quality of the growth.

## Electronic supplementary material

Additional file 1: **STROBE Statement—Checklist of items that should be included in reports of cohort studies.** (DOC 100 KB)

## Abbreviations

BPD: Bronchopulmonary dysplasia
AGA: Adequate for gestational age
SGA: For small for gestational age
IG: Intervention group
HG: Historical group.
